# Count Dracula Resurrected: Proteomic Analysis of Vlad III the Impaler’s Documents by EVA Technology and Mass Spectrometry

**DOI:** 10.1021/acs.analchem.3c01461

**Published:** 2023-08-08

**Authors:** Maria Gaetana Giovanna Pittalà, Antonella Di Francesco, Annamaria Cucina, Rosaria Saletti, Gleb Zilberstein, Svetlana Zilberstein, Tudor Arhire, Pier Giorgio Righetti, Vincenzo Cunsolo

**Affiliations:** † Laboratory of Organic Mass Spectrometry, Department of Chemical Sciences, University of Catania, Viale A. Doria 6, Catania 95125, Italy; ‡ SpringStyle Tech Design Ltd, Oppenheimer 7, Rehovot 7670107, Israel; § Sibiu County Department of Romania National Archives, Strada Arhivelor 3, Sibiu 557260, Romania; ∥ Department of Chemistry, Materials and Chemical Engineering ‘‘Giulio Natta’’, Politecnico di Milano, Via Mancinelli 7, Milano 20131, Italy

## Abstract

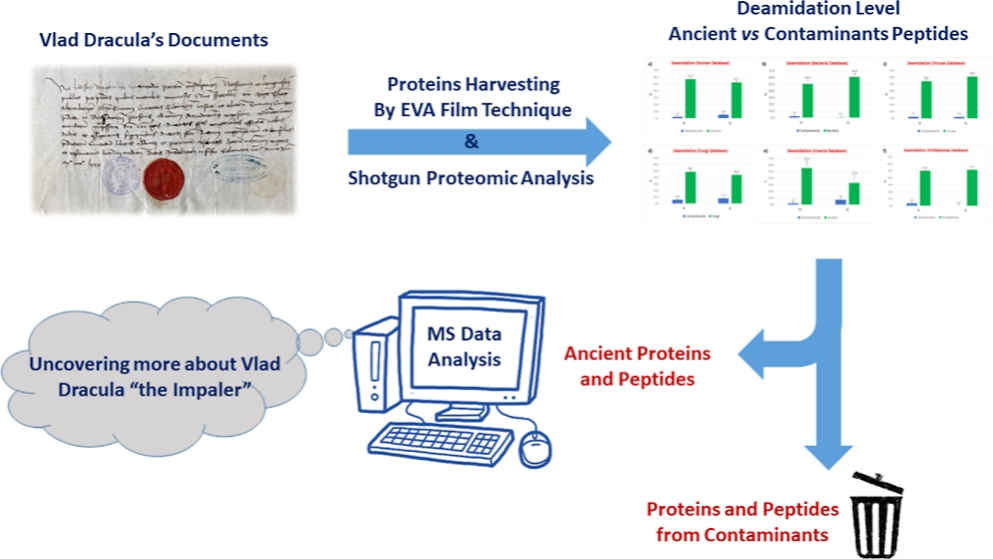

The interest of scientists in analyzing items of World Cultural Heritage has been exponentially increasing since the beginning of the new millennium. These studies have grown considerably in tandem with the development and use of sophisticated and sensitive technologies such as high-resolution mass spectrometry (MS) and the non-invasive and non-damaging technique, known under the acronym EVA (ethylene-vinyl acetate). Here, we report the results of the MS characterization of the peptides and proteins harvested by the EVA technology applied to three letters written in 1457 and 1475 by the voivode of Wallachia, Vlad III, also known as Vlad the Impaler, or Vlad Dracula. The discrimination of the “original” endogenous peptides from contaminant ones was obtained by monitoring their different levels of deamidation and of other diagenetic chemical modifications. The characterization of the ancient proteins extracted from these documents allowed us to explore the environmental conditions, in the second half of the 15th century, of the Wallachia, a region considered as a meeting point for soldiers, migrants, and travelers that probably carried not only trade goods and cultural traditions but also diseases and epidemics. In addition, the identification of many human peptides and proteins harvested from the letters allowed us to uncover more about Vlad Dracula the Impaler. Particularly, the experimental data show that he probably suffered from inflammatory processes of the respiratory tract and/or of the skin. In addition, proteomics data, although not exhaustive, suggest that, according to some stories, he might also have suffered from a pathological condition called hemolacria, that is, he could shed tears admixed with blood. It is worth noting that more medieval people may have touched these documents, which cannot be denied, but it is also presumable that the most prominent ancient proteins should be related to Prince Vlad the Impaler, who wrote and signed these letters. The data have been deposited to the ProteomeXchange with the identifier ⟨PXD041350⟩.

## Preface

The story of Count Dracula, the most famous vampire feeding on the blood of the living to live forever, is certainly one of Europe’s bloodiest legends, described by the Irish writer Bram Stoker in his book of 1897.^1^ Although the nature of this inspiration has been contentious, some scholars and historians theorized that the monstrous vampire legend in Stoker’s famous novel would partly be inspired by a historical figure: The Romanian prince Vlad III, Voivode of Wallachia during the 1400s, also known as Vlad the Impaler, or Vlad Drăculea (the Son of the Dragon).^2^ The theory that Vlad III and the Dracula of Stoker’s novel were the same person was developed and popularized by historians Radu Florescu and Raymond T. McNally in their book “In Search of Dracula” (The New York Graphic Society, 1972), but according to different historians and literary scholars, the two Draculas do not really have much in common. On the other hand, the historical figure who shares a name with the literary icon, though there is no real evidence of his alleged vampirism, was no less fearsome. This Prince of Wallachia, in the Carpathian region, reigned with terror over his territory in the 15th century. Vlad Dracula the Impaler (Vlad Tepes in Romanian) was a brutal and sadistic prince-like military leader famous for torturing his foes. Local legends say that he impaled many Ottoman enemies and a lot of people he suspected of plotting against him. By some estimates, he was responsible for the deaths of more than 80,000 people in his lifetime, a large percentage of them by impalement. Centuries later, the sinister reputation of Vlad the Impaler took on a new life when Stoker came across the name Dracula in an old history book, learned that it could also mean “devil” in Walachia, and gave the name to his fictional vampire. However, today, Vlad III is something of a national hero in Romania, where he is remembered for defending his people from foreign invasions, whether Turkish soldiers or German merchants. In light of this preface, it is undeniable that, thanks to the popularization of the image of Bram Stoker’s Dracula, Prince Vlad III himself has also become very famous and immortal, although not in the same way as his literary counterpart.

## Introduction

The interest of scientists in analyzing items (paintings, documents, ceramics, fossils, etc.) of World Cultural Heritage has been exponentially increasing since the beginning of the new millennium. Over the past two decades, studies of ancient biomolecules (mainly, but not only, proteins) have opened an important window into the past, improving our understanding, for example, of the evolutionary history,^3^ the diet habits of our ancestors,^4^ the past human diseases,^5^ or the knowledge of the materials used in a painting.^6^ These studies have grown considerably in tandem with the development and use of increasingly sophisticated and sensitive technologies and analytical tools. Particularly, the increased number of scientific publications about the characterization of ancient proteins is strictly linked to the impressive technological development, in terms of performance and sensitivity, of mass spectrometry (MS) together with the improvements of protein databases and bioinformatics tools, that has expanded the application of MS-based approaches also to the World Cultural Heritage. Modern mass spectrometers are able to detect and characterize many proteins with extremely high sensitivity even in very complex mixtures, and on very limited amounts of sample, typically less than a few micrograms of crude extract. On the other hand, it is well known that one of the most important issues of the analysis of ancient samples is represented by the contamination of exogenous proteins, mainly constituted by those coming from the post-discovery of the item or from the proteomic analysis laboratory. Therefore, the challenge of discerning contaminant proteins from endogenous ancient molecules becomes of fundamental importance. The deamidation process of asparagine and mainly of glutamine residues (showing a slower deamination rate) is the most applied marker of aging in many archaeological and paleontological studies.^7,8^ In addition, the different patterns of degradation and diagenetic chemical modifications (DCMs) affecting ancient proteins, as opposed to potential modern contaminants, are used as authentication and validation criteria for the results.^9,10^ Another important challenge is related to the inestimable value of World Cultural Heritage samples, which should be analyzed without damage or contamination. Therefore, the sampling techniques adopted have to be minimally destructive or non-destructive. Indeed, although modern high-performance mass spectrometers require very low amounts of sample, most of the actual protocols of analyses remain invasive, requiring the destruction of part of the ancient items under study; a practice that is (obviously) discouraged by most museums. Consequently, during the last decade, different minimally invasive methods have been developed to harvest and investigate ancient samples.^11−14^ A promising non-invasive technique, which at present appears to allow full exploration of any item in the Cultural Heritage arena without any damage or contamination, is known under the acronym EVA (ethylene-vinyl acetate).^15^ It consists of a plastic film of ethylene-vinyl acetate studded with strong cation and anion exchangers as well as with C18 and C8 hydrophobic resins which, when applied to any type of surface, is able to extract proteins and small molecules. Then, the harvested molecules can be eluted and characterized via MS. This non-invasive approach coupled with MS-based methods has been applied with promising results to documents, clothing items, and ancient tissue remains.^16−18^ Here, we report the results of the MS characterization of the peptides and proteins harvested by the EVA technology applied to three letters of the 15th century that were written to the burghers of Sibiu (Romania) by a man describing himself as “prince of the Transalpine regions” and signed the letters as Vlad Dracula. The characterization of proteins extracted from these three letters provided a window into Wallachia (part of today’s southern Romania) of the second half of the 15th century, a period of military turbulence, also characterized by diseases and epidemics—and not only. The identification of some human peptides and proteins harvested from the letters investigated allowed us to uncover more about Vlad Dracula the Impaler. As described by the papal legate Nicholas of Modrussy,^19^ “he was not very tall, but very stocky and strong, with a cruel and terrible appearance, a long straight nose, distended nostrils, a thin and reddish face in which the large wide-open green eyes were framed by bushy black eyebrows, which made them appear threatening.” Two green eyes that, according to some stories, sometimes cried tears of blood, a physical condition, today known as hemolacria, which causes a person to produce tears that are partially composed of blood.

## Experimental Details

### Chemicals

All the chemicals employed were used without further purification because they were of the highest purity commercially available. Ammonium bicarbonate (AMBIC), dithiothreitol (DTT), formic acid (FA), and iodoacetamide (IAA) were purchased from Aldrich (St. Louis, Missouri, USA); ammonia was purchased from Carlo Erba (Milan, Italy); sequencing grade modified porcine trypsin was purchased from Promega (Madison, WI, USA); and water and acetonitrile (ACN) (OPTIMA LC/MS grade) for LC/MS analyses were purchased from Fisher Scientific (Milan, Italy).

### Letters

**Figure 1:**
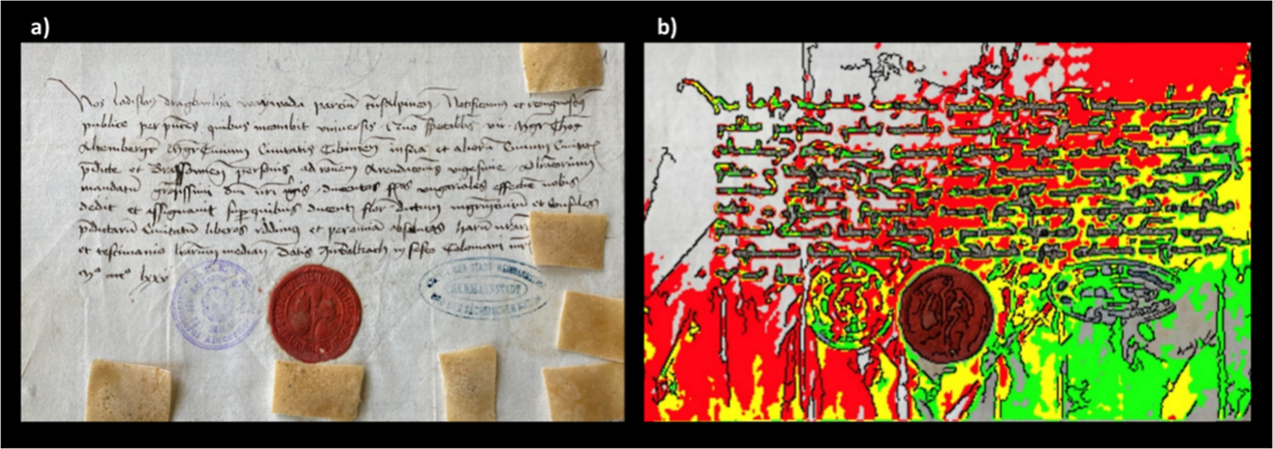
(a) First letter (archive catalog number is II 365), dated August 4, 1475, here investigated, also showing the positions of the EVA strips (brownish rectangles) applied to its surface for capturing biological material; (b) mapping of the fluorescence of phenylalanine, tyrosine, and tryptophan under flash UV illumination (see the text).

**Figure 2:**
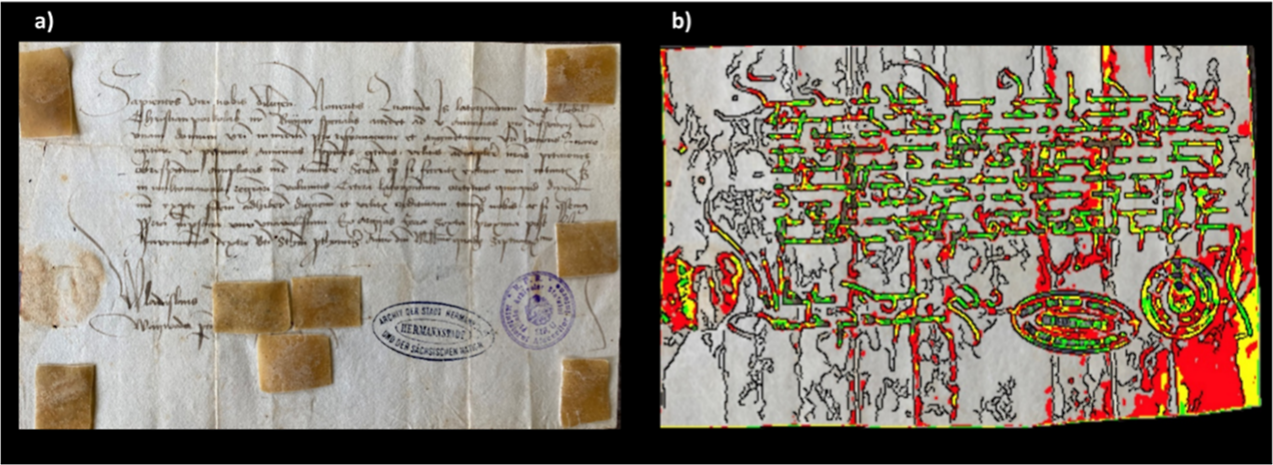
(a) Second letter (dated 1475; archive catalog number is III 32 N 484) here investigated. The letter shows the personal signature of Vlad Dracula in the left bottom part; brown patch/tape-like materials are the EVA films applied for capturing biological material; (b) mapping of the fluorescence of phenylalanine, tyrosine, and tryptophan under flash UV illumination (see the text).

**Figure 3:**
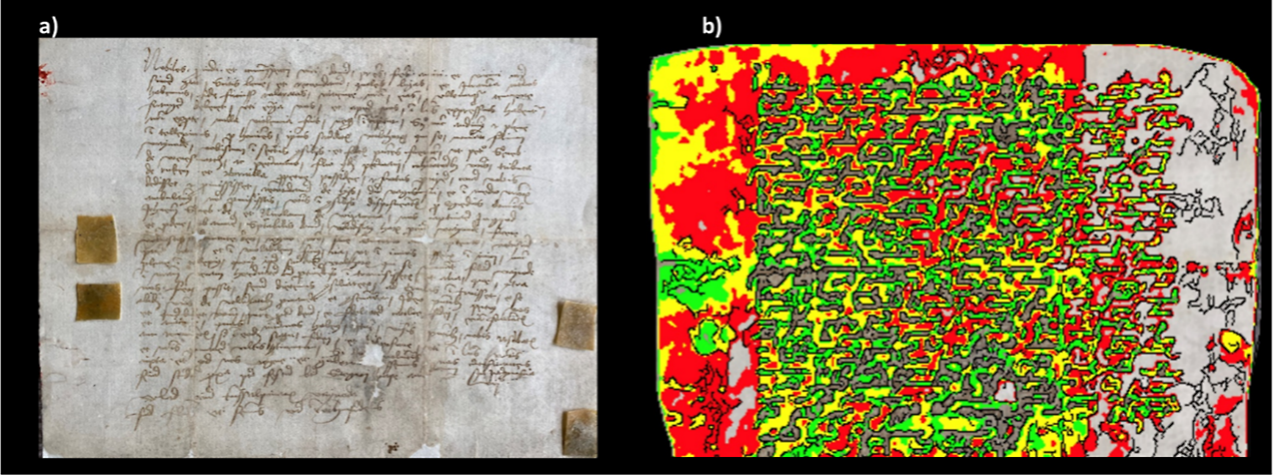
(a) Third letter (dated 1457; archive catalog number: V 1658) here investigated after restoration, and regions of sampling by EVA diskettes (the four brown patch/tape-like materials); (b) mapping of the fluorescence of phenylalanine, tyrosine, and tryptophan under flash UV illumination (see the text).

The documents investigated are three letters, made of rag paper, written and signed by the voivode of the Transalpine regions, Vladislav Dracul. Vlad Dracula wrote these three documents in different settlements of Transylvania (Bălcaciu and Braşov) and sent these documents to the city of Sibiu. Its addressees were the rulers of the city of Sibiu and, in particular, the ruler of the city of Sibiu Master Thomas Altemberger. The first letter (Figure 1a) was written in 1475 (archive catalog number is II 365); the second letter (Figure 2a) also written in 1475 (archive catalog number is III 32 N 484) and contains the personal signature of Vlad Dracula in the left bottom part; the third letter (Figure 3a) was written in 1457 (archive catalog number is V 1658). The two documents dated 1475 (Figures 1a and 2) have been kept for their entire 500 years’ history in the archives of the city of Sibiu; they are in excellent condition and were not restored. The letter written in 1457 (Figure 3a) was instead restored in Bucharest in the 20th century. All restoring operations were carried out minimizing the possibility of biological and chemical contamination. There is probably a big difference in the quality of the preservation of documents of 1475 and 1457 because the Sibiu Archive was founded in 1465 and it has been officially operating since that year. So, the two well-preserved documents of 1475 immediately began to be stored in the city’s official archive. The conditions for storing documents include storage in a paper envelope and in separate folders separately from each other. Documents can only be touched with gloves. Each view of a document is recorded on a record sheet, and the history of document views is traced. A complete set of photos of all three Dracula letters (without EVA foils), photographed on both sides, is provided in the Supporting Information. The first letter (Figure 1a), dated August 4, 1475, and translated from Latin to English, reports as follows:

“We, Vladislav Dracul, voivode of the Transalpine regions, publicly notify and recognize/by the present witnesses, who are all responsible, that the illustrious master Thomas/Altemberger, master of the people of the town of Sibiu, for himself and the other people of/said town and of the town of Braşov, in order to pay the twentieth-part [tax] as by written/command of our gracious lord and king, effectively gave and allotted to us two hundred/Hungarian florins. About those two hundred florins, we free the said master and the consuls of the/aforementioned towns, making them unencumbered and entirely released by the power and/testimony of this document. Written in Bălcaciu, in the day of St. Coloman martyr, in the year of the lord/1475.”

### Synthesis of the EVA Films and Protein Harvesting

The synthesis of the EVA film was carried out under biologically and chemically clean conditions as previously reported by Cucina et al.^20^ In particular, a special plastic-like film based on EVA as a binder of ground AG 501 mix-bed cation/anion exchange, C8 and C18 resins (all from Bio-Rad) was prepared. The mixture was made comprising 70% 1–10 μm size ground beads and 30% EVA (the melting temperature was 75 °C). This mixture of melted EVA and Bio-Rad resins was poured into a “Brabender” mixer W30 and extruded via a “Brabender” extruder KE19 (both from Brabender GmbH, Duisburg, Germany) in the form of either a thin film or diskettes. The final thickness of the EVA film was 150–200 μm. The proportion of the various resins in the plastic film was: 35% strong cation, 35% strong anion exchangers, and 15% C8 and 15% C18 hydrophobic resins. EVA foils were manufactured and supplied by SpringStyle Tech Design Ltd (Israel) with proprietary technology.

### Protein Sampling by EVA Diskettes

Protein sampling by EVA diskettes was carried out at the government archive of Sibiu, Romania, under clean conditions with nitrile gloves, tweezers, clean and sterile covers, and support surfaces. For sampling from large area letters, it is necessary to find places with a higher concentration of proteins. For this navigation, the fluorescence of phenylalanine, tyrosine, and tryptophan under UV illumination was used. Particularly, UV LED for illumination and a digital camera on a smartphone with a special optical filter for fluorescence detection were used. Special software for augmented reality interface was used for quick indication of places with high concentrations of proteins. The fluorescence level at each point was displayed in pseudo-colors on the instrument interface (green, yellow, and red, in order of increasing fluorescence intensity). This made it possible to quickly identify regions for sampling on Dracula’s letter surfaces (Figures 1b, 1c, and 1c). This mobile system and software were made in SpringStyle Tech Design Ltd for quick examination of protein traces’ presence on archaeological and culture heritage samples. To harvest proteins, the EVA diskettes were gently humidified with ultrapure water and then placed on letters in different regions for 60 min (see Figures 1a, 2a, and 3a). Finally, to prevent drying of the EVA films, they were covered with parafilm from the outside. A modern letter written and touched by the authors was used as the reference sample. Protein sampling by EVA diskettes from the modern reference letter and proteomics characterization were carried out in the same way as that of the ancient letters (see the Supporting Information).

### Protein Extraction from EVA Diskettes

EVA diskette handling and protein extraction protocol were carried out in a dedicated laboratory “clean room” in compliance with protection guidance for ancient samples and adopting all precautions to minimize the effects of contamination from modern proteins, as previously reported.^21^ A section (2 mm × 2 mm) of each EVA diskette was cut with a scalpel and put in an Eppendorf microtube. Proteins harvested in EVA films were eluted sequentially with a total of 1.3 mL of volatile buffers (formate at pH 3, followed by ammonia at pH 10, and sequentially by bicarbonate buffer at pH 8 with Rapigest 0.1%) and finally with volatile solvents (acetonitrile). The eluates were dried under vacuum (Concentrator Plus, Eppendorf) and then resuspended in 300 μL of 50mM AMBIC. Proteins were quantified by a fluorometric assay using the Qubit Protein Assay kit with the Qubit 1.0 Fluorometer.^22^ Then, about 50 μg of protein extracts (in 50 mM AMBIC solution) were reduced by 38 μg of DTT (3 h, at room temperature) and alkylated by 79 μg of IAA (1 h, in the dark at room temperature). Enzymatic protein digestion was carried out overnight at 37 °C, by 0.98 μg porcine trypsin. Tryptic peptide mixture solutions were dried under vacuum (Concentrator Plus, Eppendorf), re-dissolved in 100 μL of 5% aqueous FA, filtered by ultracentrifugation (750 μL, 0.2 μm Nonsterile Micro-Centrifugal Filters, Sepachrom, Rho, Milan), and analyzed by nano-UHPLC/high-resolution nano-ESI–MS/MS in duplicate. An empty diskette of EVA film was used as the control sample. It was processed and analyzed by proteomics in the same way as that of the EVA diskettes of the letters.

### MS Analysis

MS data were acquired via a Thermo Fisher Scientific Orbitrap Fusion Tribrid (Q-OT-qIT) mass spectrometer (Thermo Fisher Scientific, Bremen, Germany) coupled online with a Thermo Scientific Dionex UltiMate 3000 nano-Liquid chromatography system (Sunnyvale, CA). nLC-nESI MS and MS/MS analyses were carried out using the instrument parameters reported in Cucina et al.^21^ Full scans of peptide precursors were performed in high resolution (i.e., 120 K resolution @ 200 *m*/*z*), whereas tandem MS of those precursors with charge state 2–4 was carried out in the ion trap (low-resolution acquisition). To avoid cross-contamination with other biological samples, all solvents were prepared freshly, and ancient samples were not processed or analyzed in one batch with modern references. In addition, to avoid carryover during nLC-MS/MS runs, three to five blank runs were performed before each analysis using the same gradient program. Spectra acquired in the last blank run were searched by PEAKS software against the Swiss-Prot database without species origin restrictions and using the same parameters as the archaeological samples.

### Database Search

All MS data were merged and processed using two different search engines, the PEAKS X *de novo* sequencing software (v. 10.0, Bioinformatics Solutions Inc., Waterloo, ON, Canada) and the MaxQuant (MQ) software 1.6.17.0 (https://www.maxquant.org/). Raw MS data were searched against the Swiss-Prot database restricted to different taxonomies separately. Particularly, the following taxonomies were investigated: (i) “Human” (20,386 entries, release July 2022); (ii) “Bacteria” (340,707 entries, release January 2023); (iii) “Viruses” (17,957 entries, release February 2023); (iv) “Fungi” (36,956 entries, release January 2023); (v) “Insecta” (10,986 entries, release February 2023); and (vi) “Viridiplantae” (42,802 entries, release February 2023). The common Repository of Adventitious Proteins (c-RAP; https://www.thegpm.org/crap/) contaminant database was also enabled as background in all the database searches. The first step of database search was carried out using the following parameters: (a) tryptic peptides with a maximum of two missed cleavage sites; (b) cysteine carbamidomethylation as a fixed modification; and (c) oxidation of methionine, the transformation of N-terminal glutamine and N-terminal glutamic acid residue to pyro-glutamic acid form, the deamidation of asparagine and glutamine, and the N-terminal protein acetylation as variable modifications. Then, to improve peptide identification, databases were also searched investigating the following PTMs, as variable modifications: (i) oxidation, di-oxidation, formation of kynurenine, and formation of oxo-lactone, for tryptophan residues; (ii) oxidation, di-oxidation, and formation of dopaquinone, for tyrosine residues; (iii) di-oxidation of methionine; and (iv) trioxidation of cysteine. The precursor mass tolerance threshold was set to 10 ppm, and the max fragment mass error was set to 0.6 Da. Peptide spectral matches (PSMs) were validated using a Target Decoy PSM Validator node based on q-values at a false discovery rate (FDR) ≤ 0.1%. PEAKS score thresholds for PSMs were set to achieve, for each database search, FDR values for PSMs, peptide sequences, and proteins identified below the 0.1% value. In the MaxQuant software, the match type was “match from and to,” the decoy mode was “revert,” the PSM, Protein, and Site decoy fraction FDR were set at 0.01 as the threshold for peptide and protein identifications. The minimum score for modified and unmodified peptides was set at 40. All the other parameters were set as default. A protein was considered identified if a minimum of two peptides (including at least one unique peptide) were matched. Finally, to be sure of the species assigned by the software to each protein identified, all the identified peptides underwent both the BLASTp (Basic Local Alignment Search Tool for protein) searches through the NCBI database (http://blast.ncbi.nlm.nih.gov/Blast.cgi) and the “Tryptic Peptide Analysis” of the open-source web application Unipept (https://unipept.ugent.be/) to check the taxon-specificity, validate species identifications, and rule out conserved peptides between species. The MS data have been deposited to the ProteomeXchange Consortium (http://proteomecentral.proteomexchange.org) via the PRIDE partner repository with the data set identifier < PXD041350>.^23^

### Calculation of the Level of Deamidation and Other Modifications

An estimation of the percentage of deamidation for each sample was calculated with the aid of a freely available command-line script for Python 2.x (https://github.com/dblyon/deamidation), which uses the MaxQuant “evidence.txt” file.^24^ The calculations were done separately for potentially original peptides and potential contaminant peptides as previously reported.^4^ Analogously, estimation of the percentage of the other amino acid modifications investigated was obtained by applying the same model of the deamidation script, separately for potentially original and potentially contaminant peptides (see the Supporting Information).

### Metaproteomic Analysis

The web open-source application of Unipept (Unipept 4.3; http://unipept.ugent.be)^25^ was used for metaproteomic analyses. Only those peptide matches with an ion score greater than 40, assigned by Max Quant to all the peptides matched, were used in this analysis. This tool analyzes tryptic peptides, calculating the Lowest Common Ancestors (LCA) of a group of peptides, and shows the most specific taxonomic level for each peptide, giving an insight into the biodiversity of the sample and integrating complementary functional analysis.^26^ Ubiquitous peptides instead are generically assigned to “organism.”

## Results

As reported above, ancient proteins undergo a series of complex diagenetic reactions (i.e., chemical modifications of amino acids, chemical degradation, and molecular breakdown) that alter their sequence and chemical structure.^9^ Some substrates (such as bone, dental calculus, and eggshell) may preserve, from degradation driven by external factors, endogenous proteins better than others. On the contrary, because of the poor screen effect, diagenetic effects may be more extensive in substrates such as animal tissues, manuscripts, or paints. Therefore, identification of proteins may result in more challenges or even failure. In light of this issue, in the present study, we considered not only those proteins identified with a minimum of two peptides (including at least a unique peptide) but also proteomic data interpreted at the peptide level (i.e., not considering also the proteins from which these peptides come). In this way, all the “original peptides” identified by Max Quant were used to perform a *meta*-proteomics analysis by Unipept as reported in the “Materials and Methods” section to also achieve a global vision of the taxonomic distribution of all peptides. Globally, the approach adopted here allowed for the identification, as potential endogenous original components of the investigated letters, of 575 human-related peptides (Table S1), 692 peptides from Bacteria (Table S2), 389 peptides from Viruses (Table S3), 436 peptides from Fungi (Table S4), 301 peptides from Insecta (Table S5), and 394 peptides from Viridiplantae (Table S6). Instead, 165 peptides were related to proteins of the C-Rap database and therefore classified as potential contaminants (see Table S7). A modern reference letter was analyzed and processed in the same way as that used for the ancient samples. The raw data were analyzed against all the databases described in the main manuscript, and all the PTMs observed in the sample were searched. It was possible to identify: (i) 359 peptides by searching the Human database; (ii) 401 peptides by searching the Bacteria database; (iii) 237 peptides by searching the Virus database; (iv) 293 peptides by searching the Fungi database; (vi) 137 peptides by searching the Insecta database; and (vii) 299 peptides by searching the Viridiplantae database. Overall, about merely 20 peptides for each database search were in common with the ancient letters. Peptides found in both ancient letters and modern reference letters are marked with an asterisk in Tables S1, S2, S3, S4, S5, and S6 and considered as modern contaminants. The complete results of the modern reference letter are reported in Table S9.

### Level of Deamidation of Asn and Gln Residues and Other Chemical Modifications

**Figure 4:**
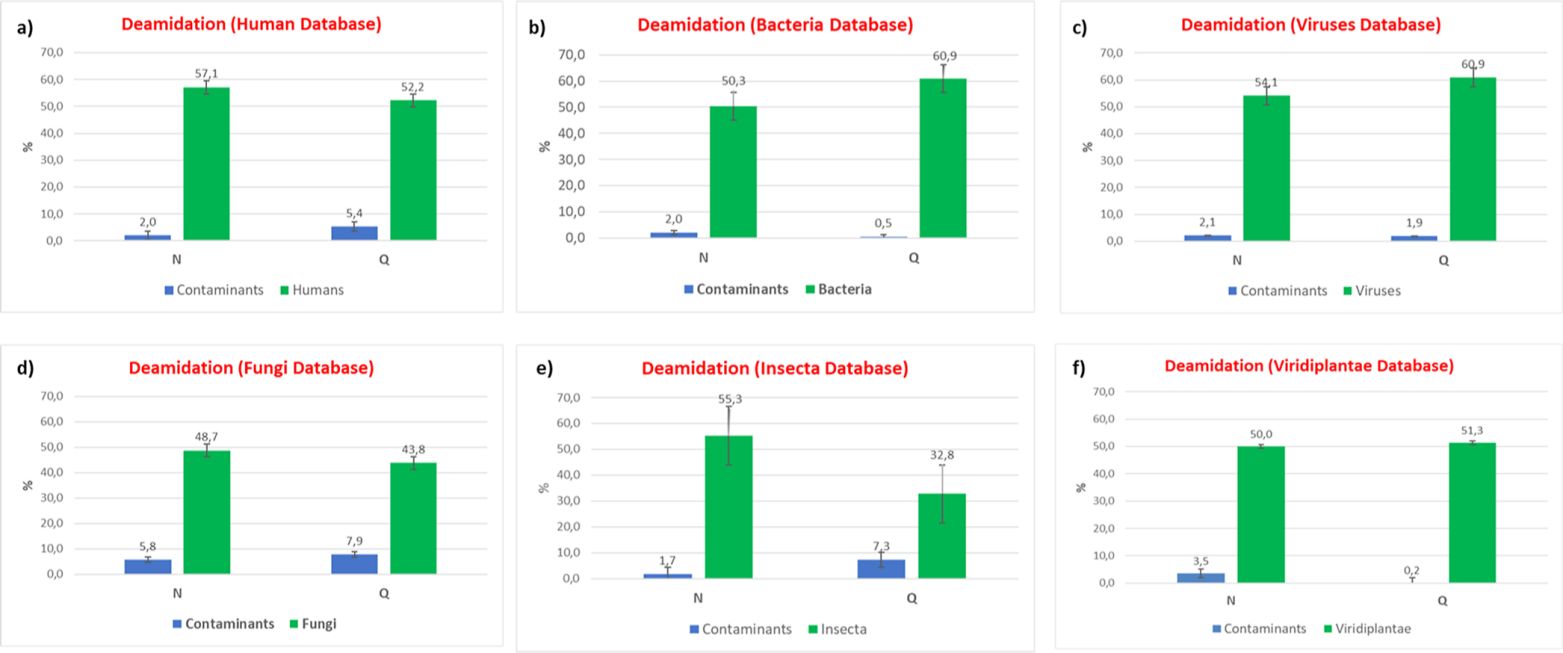
Deamidation level (reported as a percentage) of asparagine (N) and glutamine (Q) residues in “Human” (a), “Bacteria” (b), “Viruses” (c), “Fungi” (d), “Insecta” (e), and “Viridiplantae” (f) peptides. The level of deamidation of contaminant peptides identified in each database search is also reported. The error bars indicate the standard deviation calculated after 1000 bootstrap iterations.

As reported in the “Introduction” section, proteomic studies of ancient proteins in Cultural Heritage require the authentication of identified peptides and proteins to discriminate between those that genuinely originate from the sample under analysis and contaminant ones. Discrimination of the original endogenous proteins from modern contaminants may be performed by assessing the extent of degradation of proteins, taking into account that ancient proteins exhibit specific patterns of damage and modifications. In this respect, we investigated the level of deamidation (i.e., the removal of an amide group) of glutamine and asparagine residues, which are transformed into glutamic and aspartic acids, respectively, and result in a +0.98 Da mass shift.^27^ Although the deamidation rate may be affected by different environmental factors and the inherent properties of proteins,^28^ in almost all studies carried out up to date, it has been observed that it is generally much higher in ancient molecules than in modern ones.^29^ Consequently, it is one of the proposed markers of age in many archaeological and paleontological studies. In the present study, the deamidation level of asparagine and glutamine residues was calculated for all the types of peptides classified as “original” (i.e., peptides related to “Humans,” “Bacteria,” “Viruses,” “Fungi,” “Insecta,” and “Viridiplantae”). These results were compared with the deamidation level of those peptides classified as “contaminants” due to their belonging to the proteins of the c-RAP database. Figure 4 shows that the deamidation level of the “original peptides” ranges from 49 to 57% for the asparagine residues and from 33 to 61% for the glutamine residues. On the contrary, “contaminant peptides” present a deamidation level ranging from 2 to 6% for the asparagine residues and from 0.2 to 8% for the glutamine residues. These results highlight that “original peptides” show a similar deamidation level, which is about 8–28 times higher for the asparagine residues and from 5 to <100 times higher for the glutamine residues with respect to the corresponding deamidation level of “contaminant peptides.” Moreover, other DCMs may affect the sequence of the ancient proteins. These forms of random, spontaneous, and non-enzymatic alterations are mainly related to oxidative stress and damage that modify the structure of chromophoric amino acids such as tyrosine (Tyr) and tryptophan (Trp) and other amino acids such as cysteine (Cys) and methionine (Met).^30−33^ Consequently, the level of the oxidation products at tryptophan, tyrosine, cysteine, and methionine residues was calculated for both the “original” and “contaminant” peptides. Overall, the comparison of the level of the oxidation products of the above-reported amino acids in original and in contaminant peptides confirms the trend already observed for the deamidation, with the original ancient peptides having a much higher level of oxidation than the contaminant ones (see the Supporting Information and Figure S1 for details).

### Identification of the Human-Related Proteins

Among the 575 peptides from Human entries, 60 were related to 29 proteins identified with at least 2 peptides (Table S1), whereas 515 peptides came from other 515 different proteins. The 29 proteins identified with at least 2 peptides were further analyzed to validate species identifications. In particular, all the peptides that allowed the identification of these proteins underwent the BLASTp (Basic Local Alignment Search Tool for protein) searches through the NCBI database (http://blast.ncbi.nlm.nih.gov/Blast.cgi) and were also subjected to a “Tryptic Peptide Analysis” present in the open-source web application Unipept (https://unipept.ugent.be/) to check the taxon-specificity. By this search, the identified proteins were classified into three groups: (i) 16 proteins were specifically belonging to humans; these proteins were identified by at least a peptide (peptide marker) having as the lowest common ancestor the *Hominidae* family, or the Primates order; (ii) 8 proteins were related to the class of *Mammalia*; these proteins were identified with at least a peptide (peptide marker) having as the lowest common ancestor the class of *Mammalia*; and finally (iii) 5 proteins not specific because they were identified only with ubiquitous peptides. In the light of this analysis, 16 proteins were surely related to humans and were investigated by consulting the Human Protein ATLAS (https://www.proteinatlas.org/) and the literature for obtaining information about their tissue distribution and their potential involvement in diseases and pathologies. The list of these proteins is reported in Table 1. Most of these proteins were identified in all three documents, and only few differences were found among the document of 1457 and the two letters of 1475. In particular, the ETV1 and WDR74 proteins were identified only in the letter dated 1457, whereas GOLGA4, MGAM2, and KTN1 were detected only in the documents written in 1475. Many proteins show low tissue specificity, although they appear to be also related to skin (TTN, DCD, AHNAK, GOLGA4, WDR74, ZNF572, KTN1, and MDN1). Five proteins have also been found in blood (TTN, DCD, AHNAK, KTN1, and DNAH5). In particular, dermcidin (DCD) is a protein highly expressed in the sweat glands, secreted into the sweat, and transported to the epidermal surface, and has an antimicrobial activity during early bacterial or fungi colonization of *Escherichia coli*, *Enterococcus faecalis*, and *Candida albicans*, but it has also been detected in tears proteome.^34^ It should be highlighted that TTN, DCD, and GOLGA4 were also recognized, but only by a single peptide (see Table S9), in the modern reference letter. Consequently, in principle, we cannot exclude a priori these proteins from possible exogenous components. Finally, in Table 1 are reported three proteins (i.e., TRANK1, DNAH5, and DNAH11) that are mainly expressed in the airway epithelial cells and therefore related to the human breathing system. In particular, DNAH5 and DNAH11 appear to be of interest because mutations of the genes encoding these two proteins are the most common genetic causes of some ciliopathies, such as the primary ciliary dyskinesia (PCD), a collection of disorders related to cilia dysfunction that may result in retention of mucus and bacteria in the respiratory tract, and retinal degeneration.^35,36^

**Table 1:** List of Human-Related Proteins (Taxonomy Assignment after BLASTp and Unipept Analysis) Identified with at Least Two Peptides (a Detailed List of These Proteins and Related Peptides Is Rreported in Table S1)^a^

gene code	UniProt acc. no.	protein name	peptides	chemical modifications	protein tissue distribution (at the protein level)	detected in blood	detected in tears	letter of 1457 detected	letters of 1475 detected
TTN	Q8WZ42	titin	LEADVSGRPPPTMEWSKDGK	M^ox^–W^ox^	muscle tissues; skin (low)	YES		x	x
			DCIRTDGGQYILKLSNVGGTK	Y^2ox^					
			ITDQYRPK	Y^DOPA^					
TRANK1	O15050	TPR and ankyrin repeat-containing protein 1	EIESRLQLMSMDCPGQVPER	M^ox^–Deam(NQ)	many tissues (mainly in the respiratory system)			x	x
			NEIWPKMTK	W^ox-kyn^					
CENPF	P49454	centromere protein F	EISLDSYNAQLVQLEAMLRNK	M^ox^—3 Deam(NQ)	many tissues			x	x
			LSSGQNKASGK	Deam(NQ)					
DCD	P81605	dermcidin	ENAGEDPGLAR*	pGlu	skin (highly expressed in secretory cells of eccrine sweat glands)	YES	YES	x	x
			QKENAGEDPGLAR	C^TriOx^					
			YDPEAASAPGSGNPCHEASAAQKENAGEDPGLAR						
			ASAAQKENAGEDPGLAR						
			SAAQKENAGEDPGLAR						
ETV1	P50549	ETS translocation variant 1	AFPAHLPPSQSIPDSSYPMDHR	M^ox^	NA			x	
			MDGFYDQQVPYMVTNSQRGR	2 M^ox^—2 Y^DOPA^					
AHNAK	Q09666	neuroblast differentiation-associated protein AHNAK	MPDMHFKAPNISMPDVDLNLK	2 M^ox^	low tissue specificity (including skin)	YES		x	x
			VDINAPDVGVQGPDWHLKMPK						
GOLGA4	Q13439	golgin subfamily A member 4	NQEKKMEK*	M^ox^–Y^ox^	low tissue specificity (including skin)				x
			VKEAEEKILTLENQVYSMK						
MGAM2	Q2M2H8	probable maltase-glucoamylase-like protein LOC93432	LDFTLSANFQNLSLLIEQMK	M^ox^–2 Deam(NQ)	gastrointestinal tract				x
			WGGHRLGNNTAAWDQLGK	W^ox^ – W^oxolactone^					
GPRC6A	Q5T6X5	G-protein-coupled receptor family C group 6 member A	CYVIICKQEINTK	2 C^TriOx^–2 Deam(NQ) C^TriOx^–M^diox^	N.A.			x	x
			QTMFGVSFTLCISCILTKSLK						
WDR74	Q6RFH5	WD repeat-containing protein 74	SQLNCLLLSGR	C^TriOx^–Deam(NQ)	muscle tissues; skin (low)			x	
			ENALKIWDLQGSEEPVFRAK	W^ox^–pGlu					
ZNF572	Q7Z3I7	zinc finger protein 572	PYKCPDCGKSFSQSSSLIR	Deam(NQ)–C^TriOx^	low tissue specificity (including skin)			x	x
			GFSHSYVLIEHQRTHTGEK	Y^ox^					
KTN1	Q86UP2	kinectin	AQQLSITSKVQELQNLLKGK	4 Deam(NQ) pGlu	low tissue specificity (including skin)	YES			x
			ECMAGTSGSEEVKVLEHKLK						
ABCA13	Q86UQ4	ATP-binding cassette subfamily A member 13	QPSVLEAHDLKDMACVTSLIK	Deam(NQ)	N.A.			x	x
			QYGITLYSHPYGGALLNK	2 Y^DOPA^					
DNAH5	Q8TE73	dynein heavy chain 5, axonemal	VRHGMMTLGPSGAGKTTCIHTLMR	2 M^ox^–C^TriOx^	respiratory system	YES		x	x
			RTYSGVSQDLLDVSSGSQWK						
DNAH11	Q96DT5	dynein heavy chain 11, axonemal	YVPACLDKLRTSFK	C^TriOx^ pGlu	respiratory system			x	x
			QLYNEHMKQIECGHVVLNK						
MDN1	Q9NU22	midasin	QDHLWQQSTTRLTEMLKTIK	M^diox^	low tissue specificity (including skin)			x	x
			AMADFTTWK	M^ox^–W^ox^					

a Tissue distribution was obtained by consulting the Human Protein ATLAS (https://www.proteinatlas.org/) and the literature. M^ox^: methionine oxidation; W^ox^: tryptophan oxidation; Y^Diox^: tyrosine di-oxidation; Y^DOPA^: tyrosine in dopaquinone form; Deam(NQ): deamidation of asparagine or glutamine; W^ox-kyn^: oxidation of tryptophan to kynurenine; W^Oxolactone^: tryptophan oxidation to oxolactone; C^TriOx^: trioxidation of cysteine; M^Diox^: methionine di-oxidation; pGlu: Transformation of N-terminal glutamic acid or glutamine to pyro-glutamic acid. Peptides found in both ancient letters and the modern reference one are marked with an asterisk.

### Identification of the Human-Related Peptides

The remaining 515 peptides, coming from other 515 different proteins, were investigated via Unipept to check the taxon-specificity (Supporting Information Figure S2). 117 peptides (see Supporting Information Table S1) resulted specifically for Primate or sub-taxa, and therefore, they can be reasonably considered to be related to humans. Analyses by Human Protein ATLAS (https://www.proteinatlas.org/) and literature revealed that about half of these 117 peptides came from proteins having low tissue specificity. On the other hand, a group of interesting peptides is listed in Table 2. Among these 31 peptides, 11 were exclusively identified in the letter of 1457, 18 peptides were detected only in the documents written in 1475, and 2 were instead found in all the documents. Particularly, 20 peptides (7 from the letter of 1457, 11 from the letters of 1475, and 2 common to all the documents) are related to proteins detected in blood, 6 peptides (4 from the letter of 1457 and 2 from the letters of 1475) come from proteins involved in the respiratory system, whereas 3 peptides, exclusively detected in the letters of 1475, are associated with proteins of the retina and tears (GPR179, PCARE, and SAG). Moreover, it is interesting to note that 3 peptides belong to proteins (i.e., CPLANE1, from the letter of 1457, PCARE and SAG, from the letters of 1475) involved in ciliopathy or retinal diseases. Finally, 3 peptides instead belong to 3 different proteins (KLKB1, from the letter of 1457, IL17RE, and SAAL1, from the letters of 1475) involved in inflammatory processes and stimuli.

**Table 2:** List of Human-Related Peptides Coming from Proteins Detected in the Respiratory System, Blood, and Eye, Involved in Inflammatory Response, or Correlated with Ciliopathies and Retinal Diseases (a Detailed List of These Peptides Is Reported in Table S1)^a^

gene code	UniProt Acc. no.	protein name	peptide matched	observed modifications	tissue specificity (at the protein level)	detected in blood	detected in the eye (retina or tears)	letter of 1457 detected	letters of 1475 detected
ADAMTS13	Q76LX8	a disintegrin and metalloproteinase with thrombospondin motifs 13	GQYWTLQSWVPEMQDPQSWKGK	M^ox^–2 W^diox^–W^oxolactone^	respiratory system	YES		x	
ADGRG2	Q8IZP9	G-protein-coupled receptor 64	CVFWDLGRNGGRGGWSDNGCSVK	W^ox-ky^–	male tissue	YES		x	x
ALPI	P09923	intestinal-type alkaline phosphatase	HQGAWYVWNR	W^ox^–W^ox-ky^	gastrointestinal tract	YES		x	x
ASNS	P08243	asparagine synthetase [glutamine-hydrolyzing]	KYPYLWLCYNGEIYNHKK	Y^2ox^–2 Y^DOPA^	low tissue specificity	YES		x	
CMPK1	P30085	UMP-CMP kinase	IDASKSVDEVFDEVVQIFDK		low tissue specificity	YES		x	
CPLANE1	Q9H799	uncharacterized protein C5orf42	KSFGQPQGSPWPHGTATFTIQK		low tissue specificity			x	
DNAAF8	Q8IYS4	uncharacterized protein C16orf71	ALGDVPEPGAAREALMPPLEQL	M^ox^	respiratory system			x	
ELAC2	Q9BQ52	zinc phosphodiesterase ELAC protein 2	CKKEGPTLSVPMVQGECLLK	Deam(NQ)–C^TriOx^	low tissue specificity	YES			x
ENDOU	P21128	poly(U)-specific endoribonuclease	MRACISLVLAVLCGLAWAGK	2 C^TriOx^	oral mucosa; skin	YES			x
GPR179	Q6PRD1	probable G-protein-coupled receptor 179	LLQCLPCPEGCTSCMDATPCLVEEAAVLR	Deam(NQ)–2 C^TriOx^	retina—visual perception		YES		x
IL17RE	Q8NFR9	interleukin-17 receptor E	SVHFTDYSQHTQMVMALTLRCPLK	Deam(NQ)–C^TriOx^	low tissue specificity				x
IQCG	Q9H095	IQ domain-containing protein G	SREMNLEGTNLDKLPMASTITK	M^ox^–M^diox^	respiratory system				x
KIAA2012	Q0VF49	uncharacterized protein KIAA2012	PQDKNNASQHSWSLFLPK	2 Deam(NQ)	respiratory system			x	
KLKB1	P03952	plasma kallikrein	AEYRNNCLLKYSPGGTPTAIK	2 Deam(NQ)	NA	YES		x	
LRP2	P98164	low-density lipoprotein receptor-related protein 2	CVNQQCIPSR	C^TriOx^	kidney (mainly)	YES			x
MAN2B2	Q9Y2E5	epididymis-specific alpha-mannosidase	SLTGTWDLSMLHRWSWRTGPGR	M^ox^–W^ox^–W^diox^	NA	YES		x	
MMP12	P39900	macrophage metalloelastase	QMMDPGYPKLITK	M^ox^–Y^2ox^	NA	YES			x
MUC6	Q6W4X9	mucin-6	EQQEEITFKGCMANVTVTR	M^ox^–pGlu–Deam(NQ)	gastrointestinal tract	YES			x
PALLD	Q8WX93	palladin	ELQNTAVAEGQVVVLECRVR	pGlu–C^TriOx^	low tissue specificity	YES			x
PCARE	A6NGG8	uncharacterized protein C2orf71 (photoreceptor cilium actin regulator)^,^	KREPQEQPNLLQQLLQYTVSK	4 Deam(NQ)	retina—visual perception		YES		x
PLIN1	O60240	perilipin-1	TLQTTISAVTWAPAAVLGMAGR	M^ox^	male and female tissue; adipose tissue	YES			x
PLVAP	Q9BX97	plasmalemma vesicle-associated protein	ELNFTTRAKDAIMQMWLNAR	M^ox^–pGlu–Deam(NQ)	female tissue	YES			x
PPA2	Q9H2U2	inorganic pyrophosphatase 2, mitochondrial	EENGIPMKK	M^ox^–pGlu	low tissue specificity	YES			x
RNF213	Q63HN8	E3 ubiquitin-protein ligase RNF213	WQKSIVEELCAWVEKFINVK	W^ox-ky^–W^oxolactone^	low tissue specificity	YES			x
ROBO4	Q8WZ75	roundabout homolog 4	MGSGGDSLLGGR	acetyl (protein N-term)–M^ox^	adipose tissue	YES			x
ROPN1L	Q96C74	ropporin-1-like protein	WINFLALGCSMLGGSLNTALK	W^diox^	male and female tissue; respiratory system				x
SAAL1	Q96ER3	protein SAAL1	VDLPLIDSLIRVLQNMEQCQK	M^diox^	low tissue specificity				x
SAG	P10523	S-arrestin	DLYFSRVQVYPPVGAASTPTK	Y^ox^–Y^DOPA^	retina		YES		x
SCG2	P13521	secretogranin-2	QYWDEDLLMKVLEYLNQEK	pGlu–Y^ox^–Y^DOPA^	mainly endocrine tissues, gastrointestinal tract	YES		x	
SMPDL3A	Q92484	acid sphingomyelinase-like phosphodiesterase 3a	VYNAVANLWKPWLDEEAISTLRK	W^diox^–W^ox-kyn^	low tissue specificity	YES		x	
STMND1	H3BQB6	stathmin domain-containing protein 1	LLPSANHSDSAELDGAEVAFAK		respiratory system			x	

a M^ox^: methionine oxidation; W^ox^: tryptophan oxidation; Y^Diox^: tyrosine di-oxidation; Y^DOPA^: tyrosine in dopaquinone form; Deam(NQ): deamidation of asparagine or glutamine; W^ox-kyn^: oxidation of tryptophan to kynurenine; W^Oxolactone^: tryptophan oxidation to oxolactone; C^TriOx^: trioxidation of cysteine; M^Diox^: methionine di-oxidation; pGlu: transformation of N-terminal glutamic acid or glutamine to pyro-glutamic acid.

### Identification of Proteins and Peptides from the Environment

As reported above, MS data were also searched separately in five different non-human protein databases (i.e., Bacteria, Viruses, Fungi, Insecta, and Viridiplantae) to obtain a depiction of the environment related to Dracula’s letters here investigated. This database searches identified, as potential endogenous original components, 3 proteins from Bacteria, 24 from Viruses, 4 from Fungi, 17 from Insecta, and 5 from Viridiplantae. As previously reported for the human proteins, all the “environmental” proteins identified were further analyzed, via both BLASTp and Unipept searches, to check and validate the taxon-specificity. The list of these proteins, and related peptides, is reported in Supporting Information Tables S2, S3, S4, S5, and S6.

### Proteins and Peptides Related to Bacteria

Among the 692 peptides identified from the Bacteria database, 3 proteins were identified by at least 2 different peptides. These proteins belong to *Wolinella succinogenes*, a non-pathogenic proteobacteria, *Salinibacter ruber*, an extremophilic bacterium able to grow in high-salts environments, and *Mycoplasma penetrans*, a Gram-positive bacterium. *M. penetrans* is a pathogenic species that may be sexually transmitted and one cause of pelvic inflammatory disease.^37^ The remaining 686 peptides came from other 686 different bacterial proteins (Table S2). The tree-graph results of the Unipept analysis of these peptides are reported in Figure S3. Unipept analysis evidence that most of them (∼90%) are specific to the domain of Bacteria and are mainly related to the Phyla of *Proteobacteria* (∼46%) and *Firmicutes* (∼20%). Among the *Firmicutes*-related peptides, about 68% are specific to the taxonomic class of Bacilli, whereas about half (∼52%) of the *Proteobacteria*-related sequences belong to the class of gamma-proteobacteria and include the *Enterobacterales* (∼35%). Finally, it is interesting to note some peptides related to *Enterobacterales* are specific to *Yersinia pestis*, the pathogenic bacterium causing plague, whereas another group is specific to *E. coli*.

### Proteins and Peptides Related to Viruses

44 out of 389 peptides were matched by the Viruses database, and 22 different proteins were identified, mainly related to the kingdom of *Orthornavirae*. Among them, a protein (i.e., Acc. no. C5H431, see Table S3) belonged to the *Flaviviridae*, a family of viruses primarily spread through ticks and mosquitoes. Seven proteins (P36312, Q8BCV9, Q83253, P89201, Q91QZ3, Q4W382, and Q65399) are related to plant viruses, whereas the other two components (Q89709 and Q6GWS6) belong to the order of *Bunyavirales*, including viruses that may infect arthropods, plants, and vertebrates. Overall, the results of Unipept analysis of the 389 peptides are reported in Figure S4 and evidence that almost all (∼98%) are specific to the domain of Virus. About 45% of them are specifically related to the kingdom of *Orthornavirae* and mainly include specific peptides of the phyla *Pisuviricota*, *Negarnaviricota*, and *Kitrinoviricota*.

### Proteins and Peptides Related to Fungi

Searching MS data against a database of Fungi allowed us to match 436 peptides (see Table S4). Four proteins were identified with at least two peptides: three of them belonging to the class of *Saccharomycetes*, and the last related to the class of *Sordariaceae*. The Unipept analysis (Figure S5) shows that almost all of the identified peptides (∼92%) are specific to the Kingdom of Fungi and in particular may be listed into three subdivisions of the *Ascomycota*: *Saccharomycotina*, *Taphrinomycotina*, and *Pezizomycotina*. The *Saccharomycotina* and *Taphrinomycotina* peptide groups are mainly constituted by peptides specific to *Saccharomycetes* and *Schizosaccharomycetes*, respectively, and including fungi present in mature fruits but also pathogenic species for humans (e.g., *Candida glabrata* and *C. albicans*). Instead, the *Pezizomycotina*-specific peptides are mainly related to the classes of *Sordariomycetes* and *Eurotiomycetes*, two large groups including pathogenic fungi for plants.

### Proteins and Peptides Related to Insecta

301 peptides were matched by searching the Insecta database. Seventeen proteins were identified with at least two peptides, and are mostly related to the *Drosophila* genus (Table S5). Analogously, the Unipept analysis (Figure S6) allowed us to ascertain that almost all of the peptides related to the class of Insecta are specific to the *Drosophila* genus, whose members are often called “small fruit flies,” because many species usually linger around overripe or rotting fruit.

### Proteins and Peptides Related to Viridiplantae

On searching MS data against the Viridiplantae database, five proteins, with at least two peptides, were identified (Table S6). One of them (i.e., the xylanase inhibitor protein 1) is specific to *Oryza sativa*, two proteins (the transcription elongation factor SPT6 homolog and the transcription factor LHW) are related to the plant model *Arabidopsis thaliana*, and the last (the Eukaryotic translation initiation factor 4G) is specific for the tribe of *Triticeae*. In particular, the identification of proteins from the model plant *A. thaliana* could be related to the predominant presence of entries from this specie in the currently available protein databases, and a misinterpretation cannot be excluded. On the other hand, the Unipept analysis of the 394 peptides matched in the Viridiplantae database (Figure S7) revealed that among the peptides related to Viridiplantae (∼95% of total peptides), the most prominent groups are specific to the family of *Brassicaceae* (and in particular of *A. thaliana*) and of the order of *Poales* (mainly *Oryza* and *Triticeae*).

## Discussion

A MS-based shotgun approach was carried out to characterize the proteins captured by EVA diskettes from three letters written by Vlad III, voivode of Wallachia in the second half of the 15th century. The calculation of the level of deamidation and other DCMs allowed discrimination of the “original” endogenous peptides from contaminant ones. The approach adopted here permitted the identification, as potential endogenous original components, of 16 proteins that are certainly of human origin. Very few differences were identified between the letter of 1457 and the two documents dated 1475. As expected, many of the detected proteins are related to skin, but three proteins (i.e., TRANK1, DNAH5, and DNAH11) involved in the human breathing system were also identified. In particular, two breathing-related proteins (DNAH5 and DNAH11) appear of interest because mutations of the genes encoding these two proteins are involved with some ciliopathies of the respiratory tract and with retinal diseases. Moreover, five proteins (TTN, DCD, AHNAK, KTN1, and DNAH5) coming from blood were identified. In particular, a protein highly expressed in the sweat glands (i.e., dermcidin, DCD), which is also part of the tear’s proteome, was detected. In addition, taking into account the very high level of degradation of ancient proteins, proteomic data were interpreted also at peptide levels (i.e., not considering only the proteins identified by at least two peptides). In this way, we characterized about 500 peptides, of which about 100 peptides were certainly of human origin. Among this group of human peptides, 31 appear to be of interest. Many of them were detected in the documents written in 1475, whereas 11 were found only in the letter of 1457. Peptides related to blood proteins or coming from proteins involved in the respiratory system were identified in all the documents characterized. Moreover, in all three letters, some peptides related to proteins involved in ciliopathy or retinal diseases, or belonging to proteins associated with inflammatory processes, were also detected. On the contrary, three peptides associated with proteins of the retina and tears were detected only in the documents dated 1475. Although proteomics data cannot be considered exhaustive alone, altogether, these identifications might indicate that Dracula “cried tears of blood”, i.e., he suffered from the condition of hemolacria, as reported by some stories. The differences observed between the letter of 1457 and the two documents of 1475, and in particular the identification of peptides of proteins of the retina and tears only in the last two letters, may be related to the better quality of the preservation of these documents, as described above. However, a different disease state of Count Dracula cannot be excluded between 1457 and 1475. On the other hand, the identification in all three documents of some peptides coming from breathing-related proteins and/or involved with some ciliopathies of the respiratory tract, together with peptides belonging to proteins associated with inflammatory processes, might provide a picture of the general health of Count Dracula who probably also suffered from inflammatory processes of the respiratory tract and/or of the skin. It is important to highlight that we cannot deny that probably more people may have touched these documents, but it is also presumable that the most prominent protein fingerprint should be related to Prince Vlad the Impaler. Additionally, a metaproteomic investigation of the three letters permitted the identification and characterization of thousands of peptides coming from Bacteria, Viruses, Fungi, Insecta, and Viridiplantae that may help to open a window, at the molecular level, into 15th-century life. Most of the bacterial peptides come from the gut flora. Particularly, they belong to the phylum of *Firmicutes*, a Gram-positive group of bacteria, or belong to *Enterobacterales*, a large group of Gram-negative bacteria including a number of pathogens present in the human intestinal tract that are a normal part of the gut flora, but may also represent a common cause of intestinal, urinary tract infections, and also cause health-care-associated infections. It is worth noting that among the *Enterobacterales*, some peptides are specific to *Y. pestis*, the pathogenic bacterium causing plague, which led to the death of about 25 million people in Europe from 1347 to 1352, and it took 150 years for Europe’s population to overcome this disaster. Among the peptides from viruses, many of them come from viruses primarily spread through ticks and mosquitoes and are related to both plants and human-pathogenic viruses. Analogously, investigation of peptides from fungi revealed the dominant presence of specific peptides belonging to fungi present in overripe fruits and also pathogenic fungi for humans (e.g., *C. glabrata* and *C. albicans*) and plants. The possible presence of overripe fruits in the same environment as these letters may be confirmed by the identification of many peptides that are specific to the *Drosophila* genus (i.e., the “small fruit flies”), insects that linger around overripe or rotting fruit. Finally, among the proteins and peptides related to Viridiplantae, the most prominent groups are specific to the family of *Brassicaceae* and of the order of *Poales* (mainly *Oryza* and *Triticeae*).

## Conclusions

By coupling the EVA technology and high-resolution MS, an in-depth exploration of three original documents written by the Romanian prince Vlad III, also known as Vlad the Impaler, or Vlad Drăculea, was carried out. This approach allowed us to characterize about 100 ancient peptides of certainly human origin and more or less 2000 ones coming from the environment (Bacteria, Viruses, Insects, Fungi, and Viridiplantae). Altogether, proteomic data here reported, although cannot be considered exhaustive alone, might indicate that, according to some stories, he probably suffered, at least in the last years of his life, from a pathological condition called hemolacria, that is, he could shed tears admixed with blood. Additionally, he also probably suffered from inflammatory processes of the respiratory tract and/or of the skin. To our reckoning, this is the first time such research has been carried out and has helped to bring to the limelight the health status of Vlad Dracula the Impaler. It cannot be denied that more medieval people may have touched these documents, but it is also presumable that the most prominent ancient proteins should be related to Prince Vlad the Impaler, who wrote and signed these letters. Moreover, data interpretation aimed to determine the taxonomic distribution of the thousands of non-human peptides has also permitted to explore the environmental conditions of the Wallachia (a region of today’s southern Romania) in the second half of the 15th century. Wallachia was a strategic place because it acted as a meeting point for soldiers, slaves, and merchants from all over Europe and the Middle Orient. These migrants and travelers, in this era just characterized in Europe by a period of exceptionally cold climate, might have carried not only trade goods and cultural traditions but also diseases and epidemics. Overall, these results have been obtained, thanks to the high performance and sensitivity of high-resolution MS coupled with the EVA technique, a sampling method known not to contaminate or damage the documents under investigation. In this respect, it is hoped thus that the EVA methodology might be applied to several other cases of historical relevance to shed more light on important documents of our past.

## Supporting Information Available

Complete set of photos of the three Dracula letters (without EVA foils) here investigated and photographed on both sides, level of oxidative products for endogenous “potentially original” and C-Rap peptides (i.e., the “potentially contaminant” peptides), and tree-view results obtained by the Unipept analysis carried out using the identified peptides in each database search (PDF)
List of proteins and the corresponding matched peptides, identified by searching MS data against: Human (Table S1) (XLSX)
Bacteria (Table S2) (XLSX)
Viruses (Table S3) (XLSX)
Fungi (Table S4) (XLSX)
Insecta (Table S5) (XLSX)
Viridiplantae (Table S6) (XLSX)
List of the identified proteins and peptides from the c-RAP database (Table S7) (XLSX)
List of peptides identified in the control blank sample (empty EVA diskette) by searching MS data in all the databases investigated (Table S8) (XLSX)
List of peptides identified in the control blank modern letter by searching MS data in all the databases investigated (Table S9) (XLSX)
