# Supplementary material for: Count Dracula Resurrected: Proteomic Analysis of Vlad III the Impaler’s Documents by EVA Technology and Mass Spectrometry
Source: Anal Chem. 2023 Aug 8;95(34):12732–44. doi: 10.1021/acs.analchem.3c01461 (PMC10469356; doi:10.1021/acs.analchem.3c01461)
Supplement: Supplementary file 1 — ac3c01461_si_001.pdf [file ac3c01461_si_001.pdf]

Supporting Information

Count Dracula Resurrected: Proteomic Analysis of Vlad III the Impaler's Documents by EVA Technology and Mass Spectrometry

Maria Gaetana Giovanna Pittalà,[1‡] Antonella Di Francesco,[1‡] Annamaria Cucina,[1] Rosaria Saletti,[1] Gleb Zilberstein,[2] Svetlana Zilberstein,[2] Tudor Arhire,[3] Pier Giorgio Righetti,[4] and Vincenzo Cunsolo[1*]

[1] Laboratory of Organic Mass Spectrometry, Department of Chemical Sciences, University of Catania, Viale A. Doria 6, 95125, Catania, Italy

[2] SpringStyle Tech Design Ltd, Oppenheimer 7, Rehovot, 7670107, Israel.

[3] Sibiu County Department of Romania National Archives, Strada Arhivelor 3, Sibiu, 557260, Romania

[4] Department of Chemistry, Materials and Chemical Engineering ''Giulio Natta'', Politecnico di Milano, Via Mancinelli 7, Milano 20131, Italy.

[‡] These authors equally contributed to the work

The documents here investigated consist in three letters, made of rag paper, written and signed in 1457 and 1475 by the voivode of the Transalpine regions, Vladislav Dracul. Photos of all three Dracula letters (without EVA foils) and photographed on both sides as reported in Figures S1, S2, and S3, are here displayed. On the front side is the text of the letter, on the reverse side to whom this letter was intended. The letters are written in Latin and the reader of the article will be able to see Dracula's original autograph, calligraphy, the way the letter was folded and sealed with a seal.

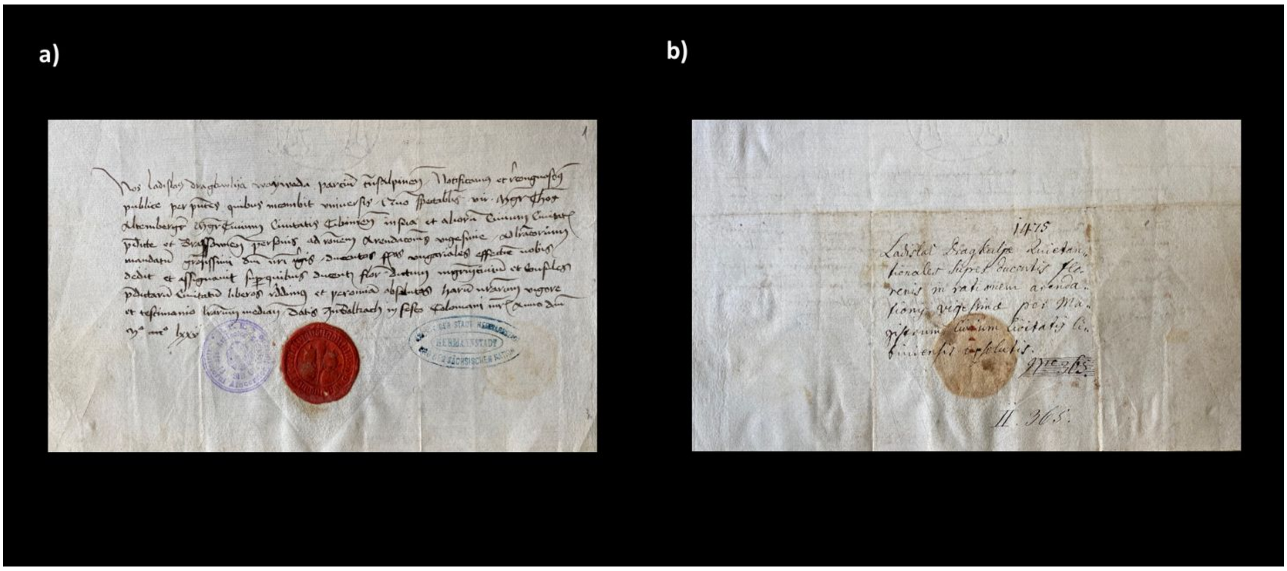

***Figure S1**. Front (a) and back (b) of the 1st letter (archive catalog number is II 365), dated August 4, 1475.*

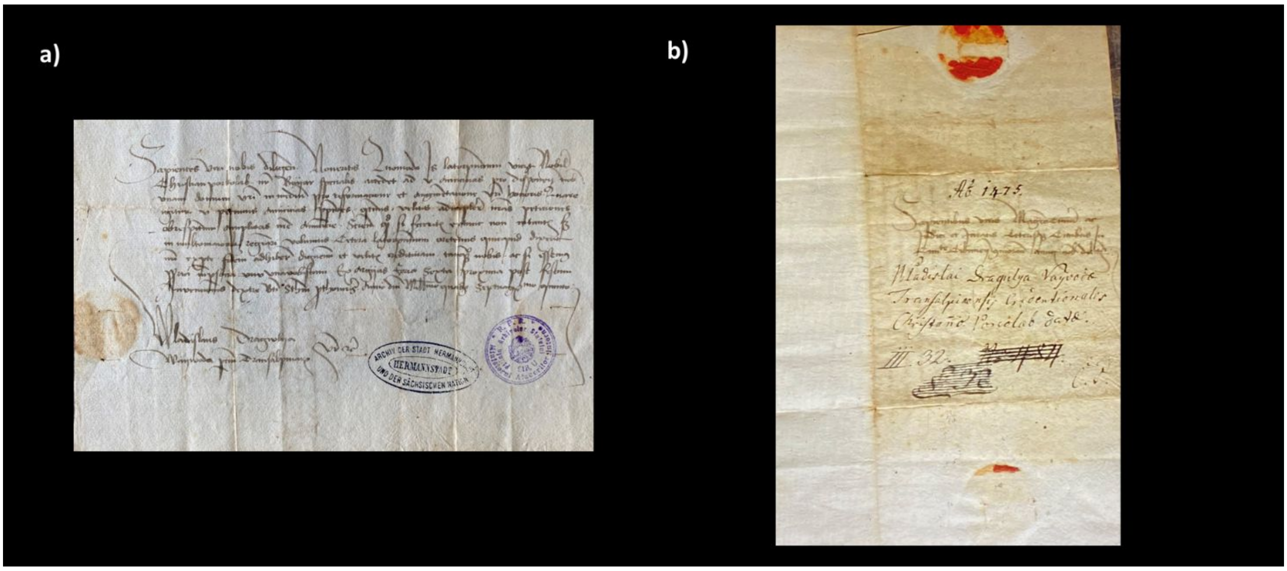

***Figure S2**. Front (a) and back (b) of the 2nd letter (dated 1475; archive catalog number is III 32 N 484) here investigated. The letter shows the personal signature of Vlad Dracula in left bottom part (a)*

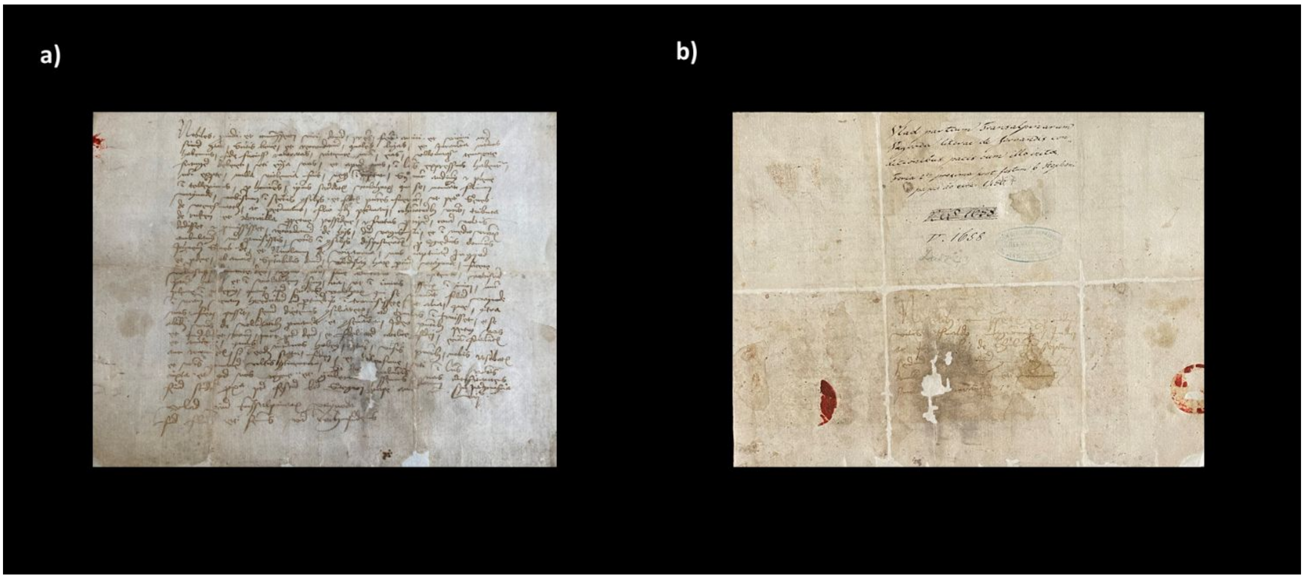

***Figure S3**. Front (a) and back (b) of the 3d letter (dated 1457; archive catalog number: V 1658) after restoration.*

The results of the research with the different PTMs were combined and the complete list of peptides and proteins are reported in the Supplementary Tables S1, S2, S3, S4, S5, S6, S7, S8, and S9 (see files excel). Also proteins identified with only one peptide are listed.

Table S1 shows the list of proteins and the corresponding macthed peptides, identified by searching MS data against "Human" database.

Table S2 exhibits the list of proteins and the corresponding macthed peptides, identified by searching MS data against "Bacteria" database.

Table S3 displays the list of proteins and the corresponding macthed peptides, identified by searching MS data against "Viruses" database.

Table S4 lists all proteins, and the corresponding macthed peptides, identified by searching MS data against "Fungi" database.

Table S5 registers the list of proteins and the corresponding macthed peptides, identified by searching MS data against "Insecta" database.

Table S6 shows the list of proteins and the corresponding macthed peptides, identified by searching MS data against "Viridiplantae" database.

Table S7 reports the list of the identified proteins and peptides from c-RAP database.

Table S8 offers the list of peptides identified in the control blank sample (empty EVA diskette) by searching MS data all the databases investigated.

Table S9 recapitulates the list of peptides identified in the control blank modern letter by searching MS data all the databases investigated.

Control blank sample: Analysis of an empty EVA diskette

An empty EVA diskette was processed in the same way of the sample. The raw data were analyzed against all the databases described in the main manuscript and all the PTMs observed in the sample were searched. About one hundred peptides could be identified. These peptides, when detected in the samples, were excluded from the final list of identifications. The results of the control blank sample are showed in Table S8 (see the corresponding file excel).

Reference modern sample: Analysis of a modern letter by EVA diskette

A modern reference letter, written and touched by the authors, was analyzed and processed in the same way of the ancient samples. The raw data were analyzed against all databases described in the main manuscript and all the PTMs observed in the sample were searched. It was possible to identify: i) 359 peptides by searching human databases; ii) 401 peptides by searching bacteria databases; iii) 237 peptides by exploring virus databases; iv) 293 peptides by searching fungi databases; vi) 137 peptides by analysing insecta database; and, vii) 299 peptides by surveying viridiplantae database. Overall, about merely twenty peptides for each database search were in common with the ancient letters. Peptides found in both ancient letters and modern reference ones are marked with an asterisk in Tables S1-S6, and are considered as modern contaminants. The results of the reference modern letter are displayed in Table S9 (see the corresponding file excel).

Calculation of the Deamidation level and the other modifications

To identify the potential contaminants introduced during sample handling and lab treatments, database searches and calculation of deamidation level were carried out using the common Repository of Adventitious Proteins (c-RAP) database (URL at ftp://ftp.thegpm.org/fasta/cRAP), a predefined contaminants database for proteomics, as background. Then, we calculated the deamidation level of asparagine and glutamine residues of potential contaminants peptides and compared it with that of potential endogenous peptides identified in the Dracula's letters.

The code for the method of the calculation is freely available to the scientific community on GitHub (https://github.com/dblyon/deamidation). The script calculation is described as follows. MaxQuant's "evidence.txt" file was used to calculate separate deamidation rates for Asparagine (N) and Glutamine (Q). The fractions of num_N (number of Asparagines) to num_N-2-D (number of deamidated Asparagines) and the fraction num_Q (number of Glutamines) to num_N-2-D (number of deamidated Glutamines) were calculated for each peptide-to-spectrum match (PSM). The values obtained were termed respectively ratio_N-2-D and ratio_Q-2-E. The ratio_N-2-D or ratio_Q-2-E was multiplied for the "Intensity" of the PTM, the values were summed and the result divided by the total sum of all intensity values of the respective unmodified peptide sequence, thus obtaining a deamidation rate between 0 and 1 for each unique peptide sequence and charge state. For each peptide an average deamidation rate for Asparagine and Glutamine was calculated. The deamidation rates were averaged per sample. The latter set of values was sampled with replacement (bootstrapped) 1000 times. The mean, the standard deviation, and the 95% confidence intervals were calculated in order to achieve an estimate of the error of the calculation.

The program generates four delimited text files as output:

- Deamidation.txt (Raw Files, deamidation for N and Q, as mean, standard deviation, 95% confidence lower and upper limit);
- Number_of_Peptides_per_RawFile.txt;
- Bootstrapped_values.txt (all the deamidation percentages calculated by e.g. 1000 bootstrap iterations, which are subsequently used to calculate the mean, std, and CI for shown in "Deamidation.txt");
- Protein_deamidation.txt (deamidation on the protein level, to be used with restraint since there usually are few data to acquire meaningful results, therefore no bootstrapping is applied).

Moreover, taking into account that proteins may be subject to damage because of exposure to light or oxidative environmental factors, many amino acids residues undergo modifications, such as oxidation, which could be index of photo-oxidative or aging damage. These forms of random, spontaneous, and non-enzymatic alterations are mainly related to oxidative stress and damage that modify the structure of chromophoric amino acids such as tyrosine (Tyr) and tryptophan (Trp), and other aminoacids such as cysteine

(Cys) and methionine (Met). Therefore, other modifications were investigated for potential contaminants peptides and compared with those of the potential endogenous peptides (see Supplementary Figure S4).

Estimation of the percentage of these modifications was obtained by applying the same model of the deamidation script, separately for potentially original and potentially contaminant peptides. In detail, for each peptide-sequence containing the residue of interest, the ratio between the number of residues in the modified form multiplied for the intensities of their peptides and the total number of the residues multiplied for the intensities of their peptides was calculated. The values obtained for each peptide (0-1) were averaged per group (potentially original and potentially contaminant peptides) and multiplied by 100 as follows:

$$PTM_{\%} = \frac{1}{n}\sum_{i=0}^{n} \frac{x_{mod} \cdot f_{mod} \cdot I_{mod}}{x_{tot} \cdot f_{tot} \cdot I_{tot}} \cdot 100$$

x = number of residues per peptide

f = number of MS scan per peptide

I = Intensity of a peptide

Results of the oxidation level in ancient and contaminants peptides

Figure S4 shows the results about the calculated oxidation level in ancient and contaminants peptides. Althought the mono-oxidation of methionine (Met) can be often an artifact of the analytical method, and the contaminant peptides may show higher values than the potential orginal ones (data not reported), the di-oxidation of this amino acid could be linked to a spontaneous aging process of the sample. In fact, the observed values of di-oxidation of methionine for potential endogenous peptides are much higher (range 29-39%) in respect to those of contaminant peptides (range 0.6-15%) (see Figure S1). Analogously, the tri-oxidation of cysteine (Cys) residues, for all groups of endogenous peptides, shows much higher values, ranging from 43 to 69%, in respect to those of peptides from contaminants (range 0-8%).

A similar trend observed for Met residues has been observed for tryptophan (Trp); indeed, if mono-oxidation level in contaminant and original peptides presents comparable values, di-oxidation of Trp for original peptides is between 12 and 31%, whereas it is always zero for contaminant peptides.

Similarly, the trasformation of Trp in kynurenine and oxolactone is higher in original peptides respect to contaminants, (see Figure S4). Finally, all the observed modifications of tyrosine (Tyr) residues (i.e. mono- and di-oxidation, and formation of dopaquinone) are remarkably higher in original peptides respect to contaminants.

Overall, these results show that original endogenous peptides present a higher level of oxidation/damage respect to the contaminant ones.

Count Dracula Resurrected: Proteomic Analysis of Vlad III the Impaler's Documents by EVA Technology and Mass Spectrometry

*M.G.G. Pittalà, A. Di Francesco, A. Cucina, R. Saletti, G. Zilberstein, S. Zilberstein, T. Arhire, P.G. Righetti, and V. Cunsolo**

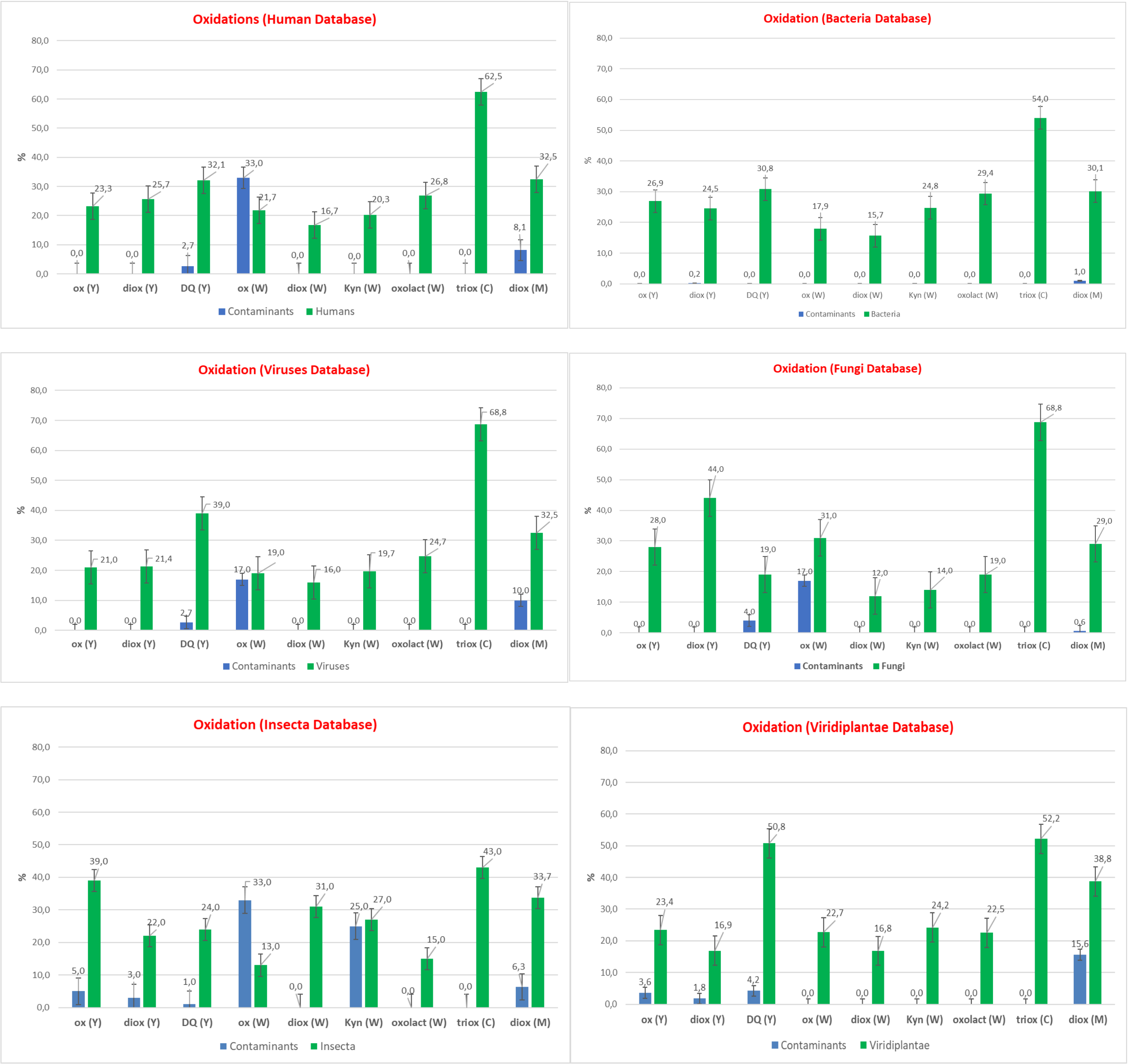

***Figure S4**. Level of modifications (oxidation, di-oxidation, and formation of dopaquinone of tyrosine residues; oxidation, di-oxidation, formation of kynerunine and oxo-lactone of tryptophan residues; tri-oxidation of cysteine residues; di-oxidation of methionine residues) for endogenous and C-Rap peptides.*

Unipept Analysis of each group of peptides identified

This tool was used to analyze tryptic peptides and calculate the Lowest Common Ancestors (LCA) for each group of peptides identified. It shows the most specific taxonomic level for each peptide, giving an insight into the biodiversity of the sample, and integrating complementary functional analysis (see the main text for details). Following the results obtained.

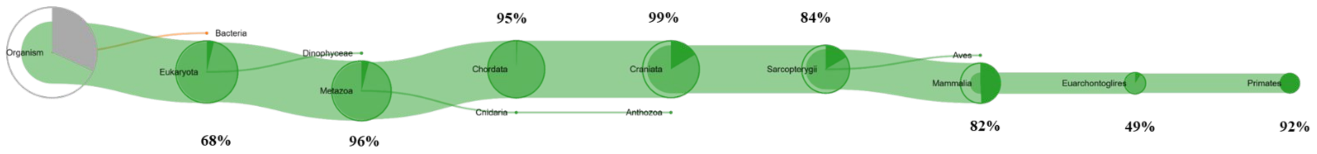

Figure S5. *Tree-view taxonomy of the peptides identified by searching the Human database. The percentage of peptides is calculated considering the 100% as the total number of peptides of the previous node. 117 peptides resulted specific for Primate and sub-taxa.*

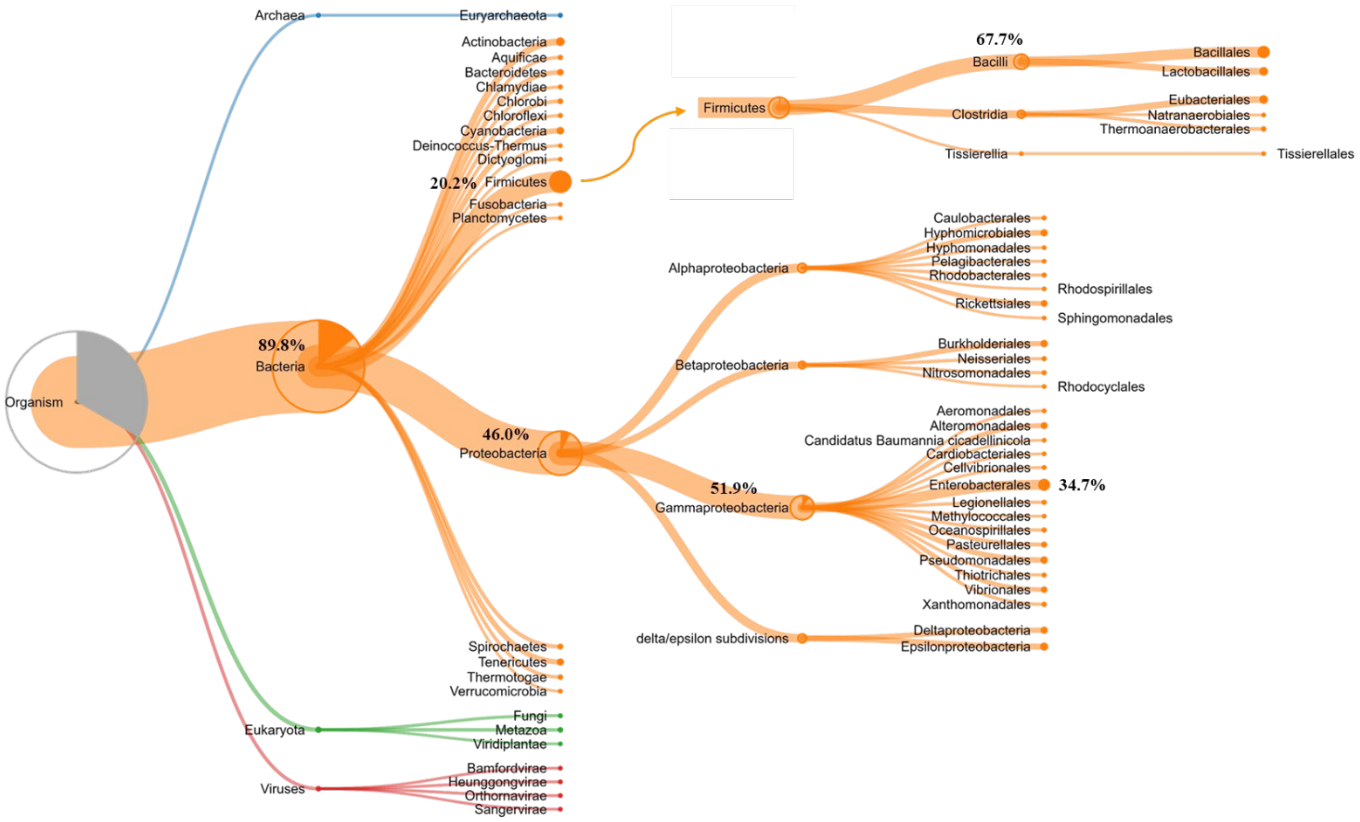

Figure S6. *Tree-view of the identified peptides by searching the Bacteria database. The percentage of peptides is calculated considering the 100% as the total number of peptides of the previous node.*

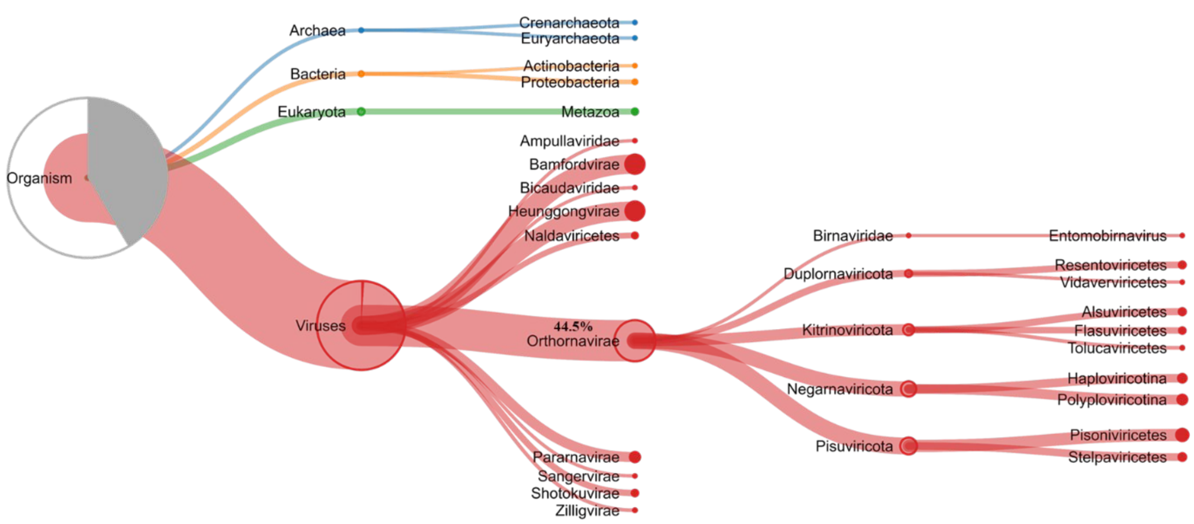

Figure S7. *Tree-view of the identified peptides by searching the Viruses database. The percentage of peptides is calculated considering the 100% as the total number of peptides of the previous node.*

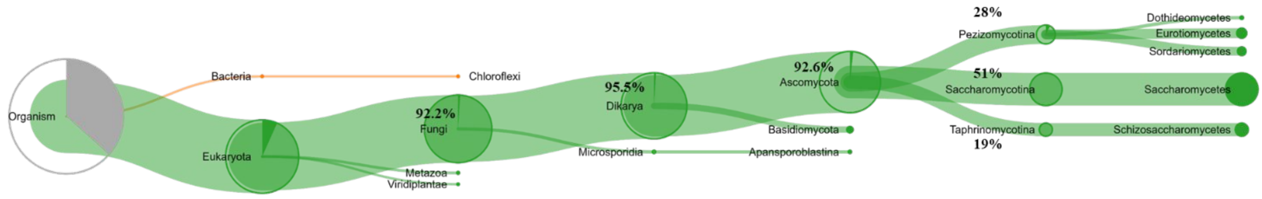

Figure S8. *Tree-view of the identified peptides by searching the Fungi database. The percentage of peptides is calculated considering the 100% as the total number of peptides of the previous node.*

Count Dracula Resurrected: Proteomic Analysis of Vlad III the Impaler's Documents by EVA Technology and Mass Spectrometry

*M.G.G. Pittalà, A. Di Francesco, A. Cucina, R. Saletti, G. Zilberstein, S. Zilberstein, T. Arhire, P.G. Righetti, and V. Cunsolo**

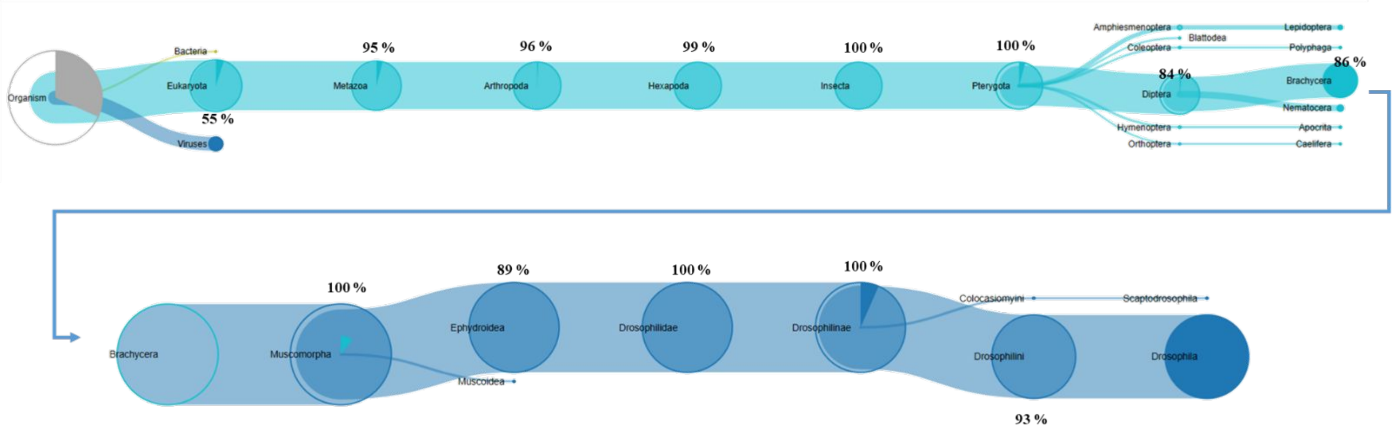

Figure S9. *Tree-view of the identified peptides by searching the Insecta database. The percentage of peptides is calculated considering the 100% as the total number of peptides of the previous node.*

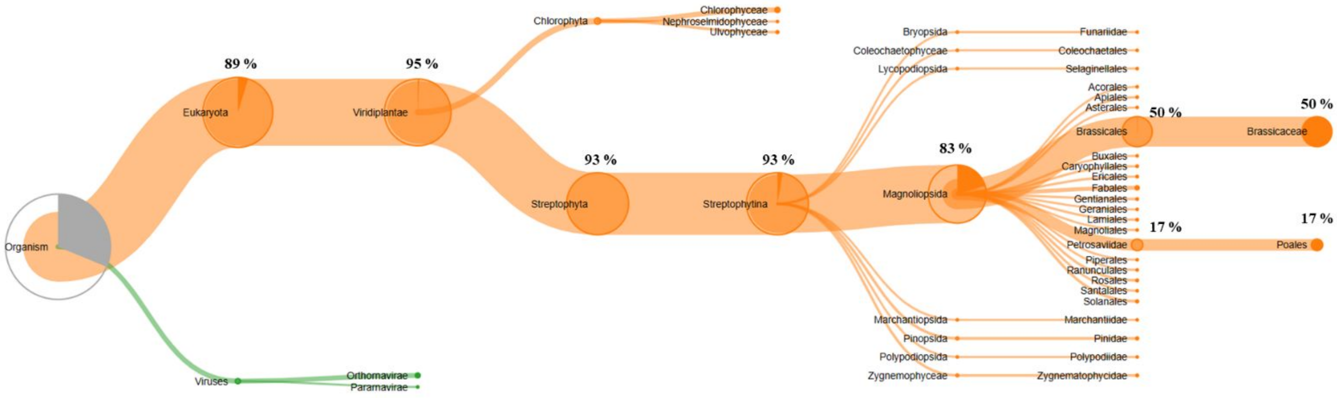

Figure S10. *Tree-view of the identified peptides by searching the Viridiplantae database. The percentage of peptides is calculated considering the 100% as the total number of peptides of the previous node*
